# Supplementary material for: Identifying tumor cells at the single-cell level using machine learning
Source: Genome Biol. 2022 May 30;23:123. doi: 10.1186/s13059-022-02683-1 (PMC9150321; doi:10.1186/s13059-022-02683-1)
Supplement: Supplementary file 1 — Additional file 1. Supplementary figures 1–4. [file 13059_2022_2683_MOESM1_ESM.pdf]

Fig S1.

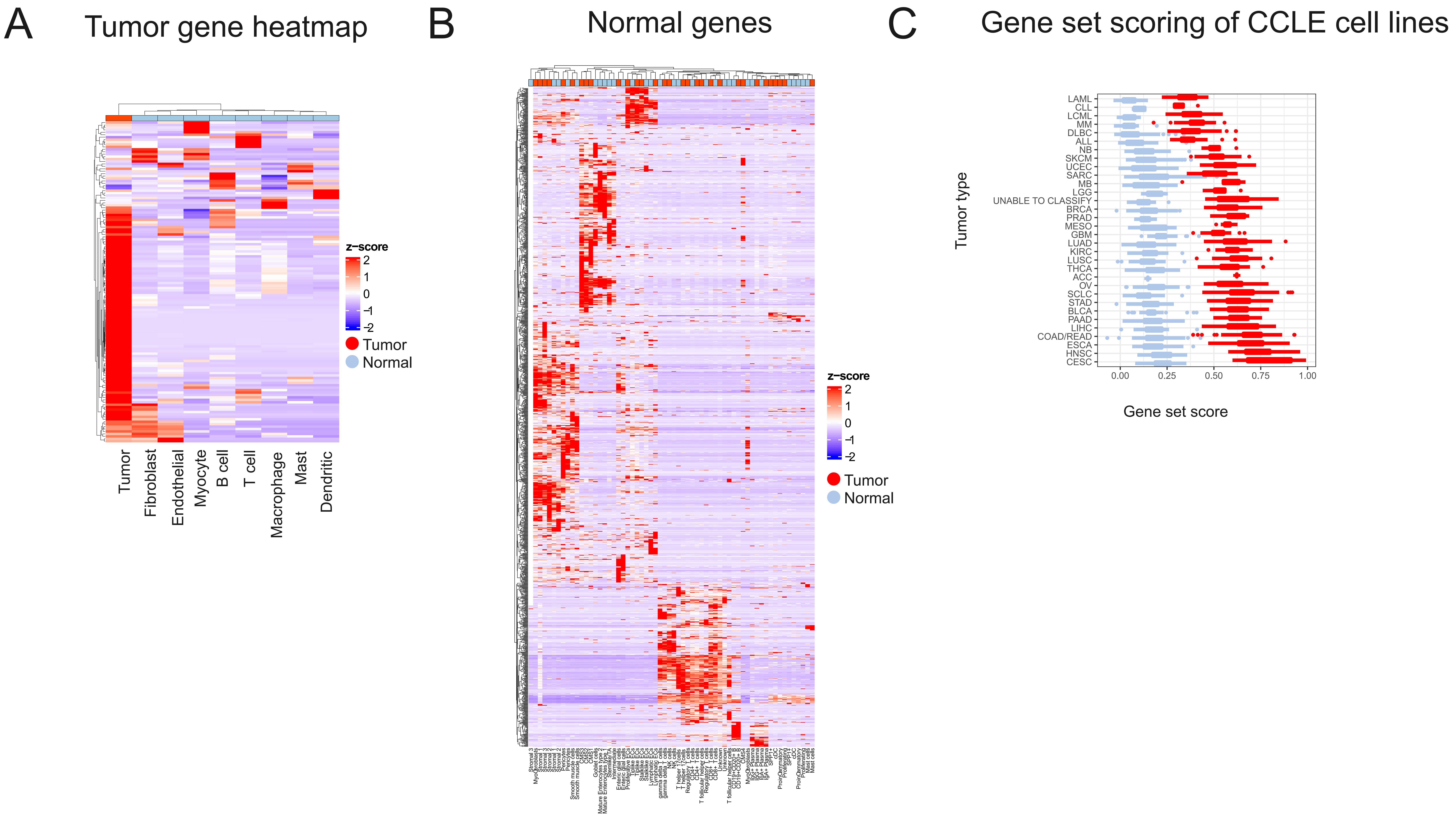

Tumor and normal gene signature characterization

A) The heat map depicts the expression of the tumor specific genes in the Tirosh head and neck cancer dataset [31], which was not used for the signature definition. Out of the 162 genes from the signature, 132 were found to be expressed in the Tirosh dataset. The tumor gene signature contains two sets of genes: genes that are highly enriched in tumor cells compared to all other cells and genes that are highly enriched in tumor cells compared to each individual cell type.

B) The normal gene signature list visualized on the Tirosh dataset (which was not used in the gene list definition). Normal gene signature captures mostly cell type specific gene expression. The tumor/normal classification designates whether the cells originated from the tumor or the healthy dataset.

C) Tumor and normal gene signature scores of the cancer cell line encyclopedia (CCLE) data. Tumor gene signature shows a significantly higher score distribution in all cancer types present in the dataset. Normal and tumor signature distributions were compared using Wilcoxon tests, for each cancer type, followed by BH-FDR correction. All adjusted p values were lower than 0.01.

Fig S2.

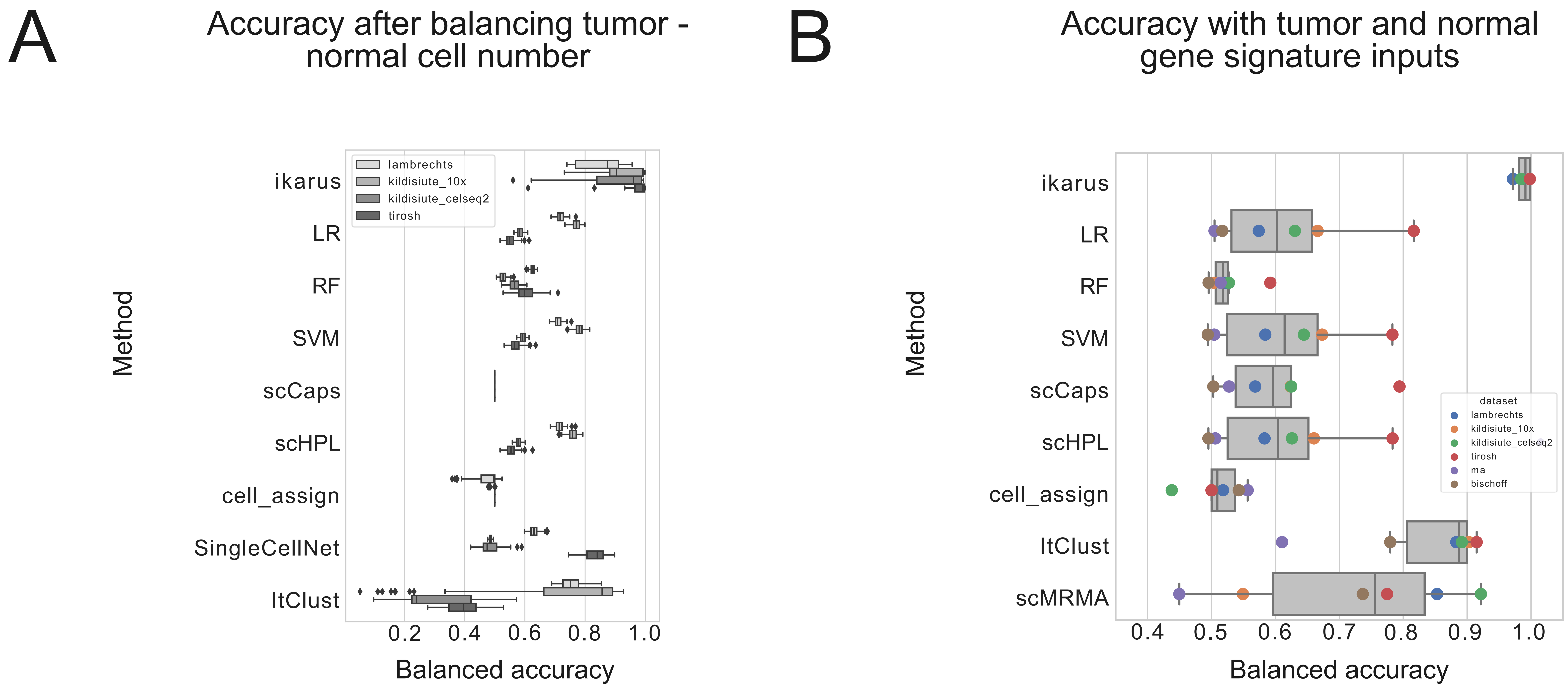

Additional tests of ikarus performance

A) Performance of ikarus and competing classifiers on datasets where the tumor and normal classes have been balanced by sampling. The sampling procedure was repeated 100 times (Distributions of results were compared using ANOVA with post hoc pairwise comparison. P values were adjusted using BH-FDR. All adjusted p values were lower than 0.01).

B) Performance of ikarus and competing classifiers when using tumor and normal gene signatures as inputs, instead of all genes. (Distributions of results were compared using ANOVA with post hoc pairwise comparison. P values were adjusted using BH-FDR. All adjusted p values were lower than 0.05).

Fig S3.

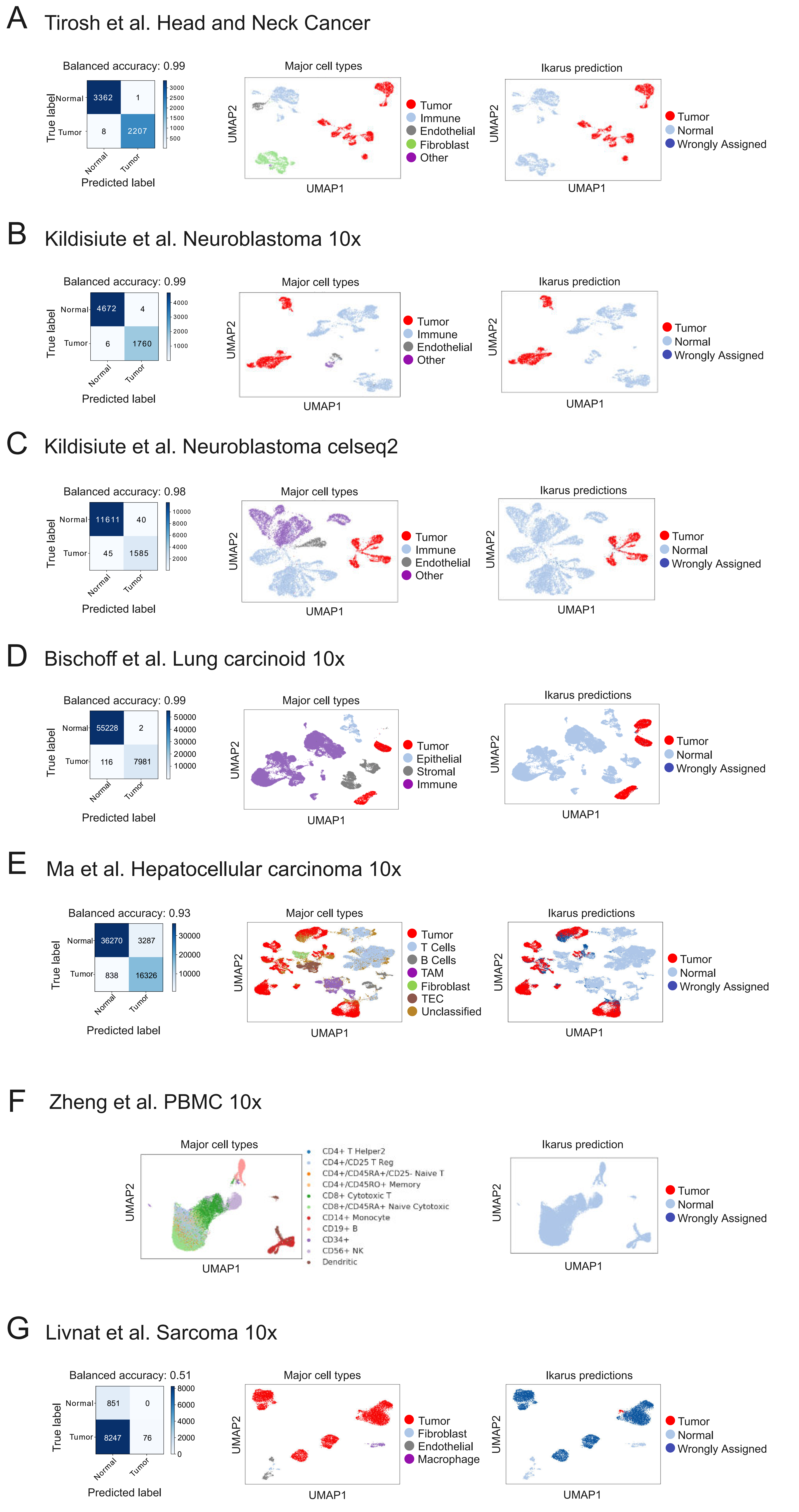

ikarus performance on multiple test datasets

A) Performance of ikarus classifier on the Puram et al. Head and Neck Cancer dataset.

B) Performance of ikarus classifier on the Kildisiute et al. Neuroblastoma dataset sequenced with 10X.

C) Performance of ikarus classifier on the Kildisiute et al. Neuroblastoma sequenced with CEL-Seq2.

D) Performance of ikarus classifier on the Bischoff et al. Lung carcinoid sequenced with 10Xx.

E) Performance of ikarus classifier on the Ma et al. Hepatocellular carcinoma sequenced with 10Xx.

F) ikarus correctly recognizes all cells from a healthy peripheral blood as non-tumorous.

G) ikarus shows a reduction in sensitivity when discrimination tumor form normal cell in a sarcoma sample [46].

Fig S4.

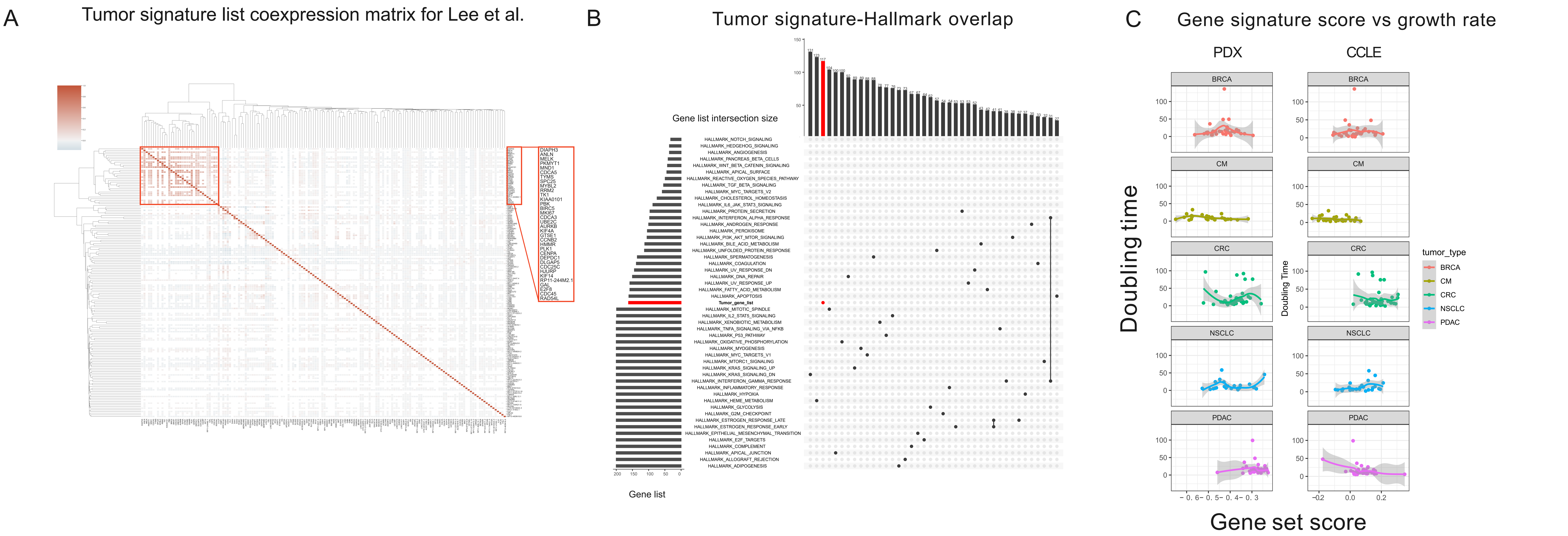

Additional annotation of the tumor gene signature

A) Correlation analysis of the tumor gene signature from Lee et al. Genes belonging to the cell cycle module are marked with a red rectangle. The comprehensive list of gene names can be found in the Table S1 - Gene signatures.

B) Tumor gene signature shows limited overlap with most of the hallmark gene sets. Only intersections of size 27 and more are shown.

C) Relationship between the tumor gene signature scores and the growth rate of PDX and CCLE models. There is limited correlation between the growth rate and the gene signatures.
