# Supplementary material for: Identifying tumor cells at the single-cell level using machine learning
Source: Genome Biol. 2022 May 30;23:123. doi: 10.1186/s13059-022-02683-1 (PMC9150321; doi:10.1186/s13059-022-02683-1)
Supplement: Supplementary file 4 — Additional file 4. Review history. [file 13059_2022_2683_MOESM4_ESM.docx]

Review History

**First round of review**

**Reviewer 1**

**Were you able to assess all statistics in the manuscript, including the appropriateness of statistical tests used?**

No

**Comments to author:**

This paper proposed a computing approach known as "Ikrus" for distinguishing tumor cells from normal cells at the single cell level. The method contains two steps, and it can mitigate two common problems in single cell analysis: the influence of batch effects on sample comparison and parameter optimization during clustering. The reviewer suggests this manuscript be thoroughly revised by fixing the following issues.
1. The abstract should summarize the main research content of the paper, and the style of the abstract of this paper is more like the introduction.
2. The main challenges of the cell annotation task of single cell sequencing data are not clearly stated. The paper should indicate the main challenges in this research area and corresponding solutions.
3. Major innovations in the paper's methodology should be shown.
4. The framework of machine learning methods should be modified so that the proposed methods can be clearly understood.
5. Unbalanced data samples could use sampling methods to process.
6. Single cell data contains lots of zeros because of dropout events. Is there any data preprocessing in the experiment?
7. As shown in the reference[1], there are many methods for single cell data analysis and cell annotation, and you can add more baseline methods.
[1] Ren Qi, Anjun Ma, Qin Ma, et al. Clustering and classification methods for single-cell RNA-sequencing data. Briefings in Bioinformatics. 2020, 21(4): 1196-1208.
8. Why is there no figure S3?

**Reviewer 2**

**Were you able to assess all statistics in the manuscript, including the appropriateness of statistical tests used?**

Yes

**Comments to author:**

The authors propose a new method, Ikarus, to classify tumor and normal cells, which has potentially broad utility. Ikarus works by deriving a gene set of interest from the data or a priori, then uses a combination of logistic regression followed by network propagation on a nearest-neighbor graph of the cells. Using these techniques, the authors propose that Ikarus is robust to batch effects when comparing samples and parameter optimization during clustering. The tool itself is available as a Python library and is uploaded to GitHub with a tutorial, along with steps to recreate figures.

The authors compare Ikarus, which was trained with a gene set derived from both a lung and a colorectal cancer data set, against other common machine learning algorithms, such as support vector machines and random forests, as well as SingleCellNet and ACTINN. They found their algorithm outperformed all other methods using balanced accuracy and AUROC measures. They continued to test the robustness of their method using a ablation analyses, removing groups or individual genes to see how the accuracy changed.

For the gene set itself, the authors identified cell cycle as the most common enriched pathway across signature databases. They did, however, identify the gene signature enriched for participation in gene fusions and copy number variation (CNV) regions. Lastly, they sought to recover the misclassified cells using CNV information gathered using inferCNV, which did improve the overall classification.

Overall, this is an interesting tool that yielded a potentially new gene signature for tumor cells. While I am interested to see this method used, there are some important ways to strengthen the manuscript. Importantly, there are almost no statistics pertaining to any comparative statement, in almost all major findings. This lowers the significance of any statement made, as they may be due to chance. It is especially important that the authors use multiple-hypothesis correction, as many tests were performed on the same data. Another important note is that the analysis of the gene set was very preliminary. Although the main focus is on Ikarus, it seems like the gene set chosen has a major impact on the classification (as seen in Figure 2). What happens with other combinations of data sets? Along this line, there was no cross-validation using a combinatorial process to combine data sets, get gene sets, and compare on the remaining data sets. While I have some reservations, I believe this is a very interesting study and would like to see an improved version. More specific details are below:

- p. 3, l. 27: Delete "The" from "The single cell sequencing technology"
- p. 3, l. 40: ikarus or Ikarus?
- p. 5, l. 33: Are these reliable labels? They appear to be unsorted. If not, then the training set may be contaminated.
- p. 6, l. 39: Why only two cancer types? Does this truly discriminate between tumor and normal? What about l. 21 where the signature was lower in blood cancers?
- p. 6, l. 9: Features were derived from these data sets, now also training on them as well? Is this an issue?
- p. 6, l. 47: Ref 8 has many algorithms, including Garnett and scID. Why were only two chosen? There should be more compared due to the number of them. Why not more specialized ones versus general classification such as SVM? Did they use the same feature inputs, the tumor vs. normal gene sets?
- p. 7, l. 7: Does accuracy change using other combinations of data sets? Are the authors suggesting using this particular gene set everywhere?
- p. 7, l. 15: This appears to be untrue. 75% of non-tumor missclassifications were immune cells. Why this bias? Is this gene set upregulated in immune cells vs. others?
- p. 7, l. 40: Ablation study very interesting!
- p. 8, l. 22: Important to show, as the first thought that comes to mind is that these are cycling cells.
- p. 8, l. 25: I would re-run these analyses without the cell-cycle related genes, especially as they are purported not to affect classification accuracy.
- p. 8, l. 38: Why so low for colorectal cancer, which the gene set was derived from?
- p. 10, l. 29: Missing space after colon.
- All subfigures have gigantic letters, they should be much smaller.
- Fig. 1: No statistics. This appears throughout the manuscript and in more places that I do not explicitly list.
- Fig. 2: No statistics.
- Fig. 2D: I see no Epithelial cells or Other cells (blue or purple), and the brown color is not in the legend.
- Fig. 3A: No statistics.
- Missing S3
- Fig. S4: Too small, cannot read or interpret most of this figure.
- I was unable to build Ikarus with the error "ModuleNotFoundError: No module named 'numpy'" from pyarrow, even though numpy is installed and is able to be loaded. To fix this, I must use "pip install -e . --no-use-pep517 --no-build-isolation", but this results in hanging with no progress on Python 3.9.6.
- The code itself had very few comments to help facilitate open-source collaboration.

**Authors Response**

**Point-by-point responses to the reviewers’ comments:**

## Reviewer #1:

This paper proposed a computing approach known as "Ikrus" for distinguishing tumor cells from normal cells at the single cell level. The method contains two steps, and it can mitigate two common problems in single cell analysis: the influence of batch effects on sample comparison and parameter optimization during clustering. The reviewer suggests this manuscript be thoroughly revised by fixing the following issues.

1. The abstract should summarize the main research content of the paper, and the style of the abstract of this paper is more like the introduction.

*We have changed the abstract to contain a more direct description of the contents of the paper.*

2. The main challenges of the cell annotation task of single cell sequencing data are not clearly stated. The paper should indicate the main challenges in this research area and corresponding solutions.

*We have incorporated, in the introduction, a more precise problem statement, along with the currently available solutions.*

3. Major innovations in the paper's methodology should be shown.

*The final paragraph of the introduction now clearly states the major innovations of the manuscript.*

4. The framework of machine learning methods should be modified so that the proposed methods can be clearly understood.

5. Unbalanced data samples could use sampling methods to process.

*We have tested Ikarus and the competing methods with data where the classes have been subsampled to contain the same number of instances - 1000 tumor and normal cells. The subsampling procedure has been repeated 100 times.*

*The following figure shows the effect of subsampling on the performance of classical machine learning classifiers, and specialized tools for cell type annotation.*

*Subsampling improves the performance of classical machine learning methods. Support vector machines and logistic regression being tied for the second place. Even with subsampled data Ikarus still achieves the highest median performance. We have seen, though, that the accuracy becomes variable. This is because the subsampling reduces the comprehensiveness of the cell - cell network which is used for network propagation. The results are now included as Figure S2.*

6. Single cell data contains lots of zeros because of dropout events. Is there any data preprocessing in the experiment?

*Raw data is normalized to log counts per million reads (logCPM), as customary for the analysis of single cell datasets. The transformation option is implemented as part of the python package. We have made the statement about normalization more explicit, both in the methods section and in the jupyter notebook tutorial on github*

*Gene signature based scoring, followed by network propagation successfully mitigates the differences in mRNA capture rates (e.g. abundances of zero values) in different cells, which otherwise cause problems during classification.*

7. As shown in the reference[1], there are many methods for single cell data analysis and cell annotation, and you can add more baseline methods.

[1] Ren Qi, Anjun Ma, Qin Ma, et al. Clustering and classification methods for single-cell RNA-sequencing data. Briefings in Bioinformatics. 2020, 21(4): 1196-1208.

*At first we have limited the comparison to the top ranking methods from the recent review* [*(Abdelaal et al. 2019)*](https://paperpile.com/c/97637O/J7iY)*. In earnest, we have tried to run as many possible methods, but for a lot of methods we either had problems with software execution (the methods did not work), or software installation.*

*We have now compared ikarus with an extended list of cell type classification methods, for all of the datasets used in the original manuscript - ItClust* [*(Hu et al. 2020)*](https://paperpile.com/c/97637O/WFDP)*, scCaps[(Wang et al. 2020)](https://paperpile.com/c/97637O/sYRB), scHPL* [*(Michielsen, Reinders, and Mahfouz 2021)*](https://paperpile.com/c/97637O/DYn0)*, CellAssign* [*(Zhang et al. 2019)*](https://paperpile.com/c/97637O/oNrQS) *from scvi-tools* [*(Zhang et al. 2019)*](https://paperpile.com/c/97637O/oNrQS)*, scMRMA* [*(Li et al. 2022)*](https://paperpile.com/c/97637O/Z3FAl)*. The methods have been also compared with the subsampled versions of the data sets where the classes have been balanced.*

*The following figure shows ikarus performance compared with an extended set of competing methods. The figure replaced Figure 2A.*

8. Why is there no figure S3?

*Figure S3 originally represented a table, which has been converted to Supplementary Table S1 - Effects of SAA1 and FGB. The label in the text was a typographic error, which we have corrected.*

*We have constructed a new Figure S3, from previous figure S2 (containing classification performance). New figure S2 contains the perturbation experiments suggested by the reviewers.*

## Reviewer #2:

The authors propose a new method, Ikarus, to classify tumor and normal cells, which has potentially broad utility. Ikarus works by deriving a gene set of interest from the data or a priori, then uses a combination of logistic regression followed by network propagation on a nearest-neighbor graph of the cells. Using these techniques, the authors propose that Ikarus is robust to batch effects when comparing samples and parameter optimization during clustering. The tool itself is available as a Python library and is uploaded to GitHub with a tutorial, along with steps to recreate figures.

The authors compare Ikarus, which was trained with a gene set derived from both a lung and a colorectal cancer data set, against other common machine learning algorithms, such as support vector machines and random forests, as well as SingleCellNet and ACTINN. They found their algorithm outperformed all other methods using balanced accuracy and AUROC measures. They continued to test the robustness of their method using ablation analyses, removing groups or individual genes to see how the accuracy changed.

For the gene set itself, the authors identified cell cycle as the most common enriched pathway across signature databases. They did, however, identify the gene signature enriched for participation in gene fusions and copy number variation (CNV) regions. Lastly, they sought to recover the misclassified cells using CNV information gathered using inferCNV, which did improve the overall classification.

Overall, this is an interesting tool that yielded a potentially new gene signature for tumor cells. While I am interested to see this method used, there are some important ways to strengthen the manuscript. Importantly, there are almost no statistics pertaining to any comparative statement, in almost all major findings. This lowers the significance of any statement made, as they may be due to chance. It is especially important that the authors use multiple-hypothesis correction, as many tests were performed on the same data.

*We have now included statistical tests for all gene set comparisons, along with multiple testing corrections. The procedures are described in the methods section of the manuscript.*

*The statistical analysis corroborates the majority of the claims from the original manuscript, due the large size effects and large N.*

*The comprehensive statistical results can be found in the Supplementary Table 2 - Statistical Comparisons, and the results are reported in the figure legends.*

Another important note is that the analysis of the gene set was very preliminary. Although the main focus is on Ikarus, it seems like the gene set chosen has a major impact on the classification (as seen in Figure 2). What happens with other combinations of data sets? Along this line, there was no cross-validation using a combinatorial process to combine data sets, get gene sets, and compare on the remaining data sets.

*This is a very important analysis which was previously absent from the manuscript, due to the scarcity of the available datasets. We have now included a cross validation procedure for gene set selection. We have used a combination of Lee, Laughney, Lambrechts, Tirosh, and Kildisiute datasets, to conduct a cross validation analysis. For each pair of datasets, gene signature selection was performed, followed by training of the logistic classifier. The resulting classifier accuracy was validated on the three datasets that were not used for training. The accuracy of the top performing classifiers was furthermore tested on the newly acquired hepatocellular* [*(Ma et al. 2021)*](https://paperpile.com/c/97637O/oZoS) *carcinoma and carcinoid datasets* [*(Jerby-Arnon et al. 2021)*](https://paperpile.com/c/97637O/LJS3) *(Supplementary Table 2 - Crossvalidation Results). As the performance metric we have chosen a minimal balanced accuracy on the validation set (i.e. what is the worst performance of the classifier on the validation set).*

*For comparison, we have also trained classifiers on gene lists extracted from each of the datasets.*

*The following figure shows the minimal balanced accuracy for every training data set combination. X axis shows the number of datasets used for signature selection, while the Y axis shows the minimal balanced accuracy.*

*The figure shows that the intersection of multiple datasets enables better generalization than using just a single dataset.*

*The training data used in the original manuscript was the combination of lee and laughney, which implies that the student who initiated the project was extremely lucky when choosing datasets.*

*Out of curiosity, we have also tested a combination of three datasets for training, and it achieved very good classification performance on both the validation and test sets (minimal accuracy of 0.9, with median accuracy of 0.96). To extend the training analysis to higher order combinations, we really need more datasets - using just two datasets for validation, and parameter selection gives low guarantees for generalization.*

While I have some reservations, I believe this is a very interesting study and would like to see an improved version. More specific details are below:

- p. 3, l. 27: Delete "The" from "The single cell sequencing technology"

*Deleted*

- p. 3, l. 40: ikarus or Ikarus?

*We have adopted a uniform labeling through the manuscript, and the github repository: ikarus*

- p. 5, l. 33: Are these reliable labels? They appear to be unsorted. If not, then the training set may be contaminated.

*To our surprise, a marker based sorting experiment for isolation of human cancer cells, followed by single cell sequencing is not a common experimental procedure (we have managed to find only one example from a head and neck cancer* [*(Puram et al. 2017)*](https://paperpile.com/c/97637O/1Xj7)*. We have therefore hypothesized that by intersecting data from independent datasets, where we do not know the contamination status of cell annotations (but the annotation errors are likely orthogonal between the studies), might produce a robust signature, and enable the classifier to generalize. By comprehensively testing the signature on laser microdissection data, cell lines, PDX models, and different types of cancers (colorectal, head and neck - where the cancer cells were sorted, neuroblastoma, hepatocellular carcinoma, and a lung carcinoid), we have seen that it does a surprisingly good job at discriminating normal from tumor cells/samples..*

- p. 6, l. 39: Why only two cancer types? Does this truly discriminate between tumor and normal?

*We have used only two datasets for training, because of the scarcity of available data when the project was initiated. Namely, we could find only six datasets which contained cells apriori classified as tumor and non-tumor. We sincerely wanted to include only datasets where the labeling was done by independent experts (and not us). In addition, our preliminary validations on laser microdissection dataset, cell lines and patient derived xenograft models all showed the same direction of the effect - same difference in the score distributions across multiple cancer types. This gave us confidence that the gene set does provide discriminatory information which might generalize across cancers with different origins.*

*We have tested the method on additional datasets, and are confident that it performs well on carcinomas.*

*The following figure shows ikarus performance on the hepatocellular carcinoma datasets:*

*The following figure shows the UMAP embeddings of cell labels, and ikarus classification results*

*In addition we have tested ikarus on a lung carcinoid sample, where it showed consistently good performance:*

*The following figure shows the confusion matrix for the lung carcinoid classification:*

*And the corresponding UMAP embeddings:*

*Both figures have been added to Figure S2.*

*We have also started to examine ikarus performance on spatial sequencing datasets.*

*The following figure shows the performance on a breast cancer sample profiled using 10x Visium platform. The left image shows cancer region annotation by a clinical pathologist, while the right shows ikarus score. The work is still preliminary, and not ready for publication but it just provides additional evidence that the work has multiple applications and we have been looking at other available datasets which we have described above.*

*We have however also found a limitation of the method. When tested on two synovial sarcoma samples, the sensitivity of the tumor signature was reduced (due to the low overlap with the tumor gene signature), and therefore the results showed a high false negative rate.*

*The following figure shows ikarus performance on the synovial sarcoma dataset*

*The following figure shows the UMAP embeddings of cell labels, and ikarus classification results*

*We have now explicitly stated the limitations of the method in the manuscript, and are actively working on expanding the functionality on additional cancer types. The figures have been included in Figure 2S.*

What about l. 21 where the signature was lower in blood cancers?

*We have seen that the tumor gene signature is highly enriched in genomic regions that are recurrently amplified in multiple cancer types (Figure 5. F), therefore we are highly convinced that the tumor gene signature score actually indirectly measures the amount of copy number variation in the cancer cells.*

*Blood derived cancers are primarily driven by recombination events (i.e. gene fusions), unlike soft tissue cancers where the primary genetic aberration is the loss or gain of large genomic regions (i.e. copy number variation), which would then explain why the gene sets scores were significantly lower in blood cancers, since they contain fewer recurrent copy number variations.*

- p. 6, l. 9: Features were derived from these data sets, now also training on them as well? Is this an issue?

*We do not believe this is an issue. In machine learning methodology it is important that all of the parameters are derived from the training set; that the defined parameter set is immutably fixed, and finally tested on the test set, without further parameter tuning. The parameters in our model consist of the gene signatures and the learnable parameters of the logistic regression. Because all of the parameters are derived from the training sets, fixed, and then tested on the four test sets; information leakage from the test data was not possible. We have tested the model on two additional datasets which were not used in the original manuscript, where it again showed very good performance.*

- p. 6, l. 47: Ref 8 has many algorithms, including Garnett and scID. Why were only two chosen? There should be more compared due to the number of them. Why not more specialized ones versus general classification such as SVM? Did they use the same feature inputs, the tumor vs. normal gene sets?

*As replied to reviewer #1. In earnest we have tried to compare ikarus with the maximal possible number of available methods, but for the majority of the methods we have experienced execution related issues. We have therefore decided to focus on the top performing classification algorithms from the* [*(Abdelaal et al. 2019)*](https://paperpile.com/c/97637O/J7iY) *- an extensive comparison of cell type classification methods. Surprisingly, the top performing method from* [*(Abdelaal et al. 2019)*](https://paperpile.com/c/97637O/J7iY) *was an SVM classifier.*

*We have now extended the comparison by including multiple additional cell classification methods, which were developed after the Abdelaal et al. review. ItClust* [*(Hu et al. 2020)*](https://paperpile.com/c/97637O/WFDP)*, scCaps[(Wang et al. 2020)](https://paperpile.com/c/97637O/sYRB), scHPL* [*(Michielsen, Reinders, and Mahfouz 2021)*](https://paperpile.com/c/97637O/DYn0)*, CellAssign* [*(Zhang et al. 2019)*](https://paperpile.com/c/97637O/oNrQS) *from scvi-tools* [*(Zhang et al. 2019)*](https://paperpile.com/c/97637O/oNrQS)*, and scMRMA* [*(Li et al. 2022)*](https://paperpile.com/c/97637O/Z3FAl)*. scMRMA is the most recent development in the gene signature based classifiers, and beats Garnett in direct comparison.*

*The figure shows the comparison of ikarus performance with additional classification methods.*

*ikarus still achieves the top performance.*

“Did they use the same feature inputs, the tumor vs. normal gene sets?”

*In the original manuscript we have used all available genes when testing competing algorithms. We have now rerun the analysis with just the tumor and normal gene signatures. The following figure shows the performance of competing algorithms with just the tumor/normal gene signatures as input genes:*

*To our surprise, reducing the input genes to the tumor/normal gene signatures improved the performance of all competing methods, with ItClust achieving a high accuracy of 0.9.*

*The increase in classification accuracy upon the input gene set reduction indicates that the tumor/normal gene signatures really do contain the information necessary for discrimination between the tumor and normal cells.*

*We have now included the figure as Figure S2B.*

- p. 7, l. 7: Does accuracy change using other combinations of data sets? Are the authors suggesting using this particular gene set everywhere?

*As stated above, we are quite convinced that the signature works well in carcinomas, and tissues ontogenetically connected with the epithelium (neuroblastomas are derived from neurons which are derived from the ectoderm, while lung carcinoids stem from neuroendocrine cells). ikarus has reduced sensitivity in sarcomas (Figure S2). Based on the signature performance on the PDX and CCLE cell lines, we believe it will work also on adenocarcinomas, it is however still an open question whether it will work on other types of glandular tumors, lymphomas, and germ cell tumors. We are continuously looking for available datasets to update the trained models to novel cancer types and validate the previously trained models.*

- p. 7, l. 15: This appears to be untrue. 75% of non-tumor misclassifications were immune cells. Why this bias? Is this gene set upregulated in immune cells vs. others?

*We have tested the abundance of misclassified cell types using a Fisher’s test., by executing a pairwise comparison of the relative frequencies of every misclassified cell type in the Lambrechts’ datasets.*

*Immune cell misclassifications are more prevalent than the misclassification of epithelial cells and the endothelial cells, but are not significantly different with respect to the fibroblast population. We have changed the text accordingly.*

| *cell types* | *p.value* | *test* |
| --- | --- | --- |
| *Immune vs Epithelial* | *0.0003* | *2x2 fisher exact* |
| *Immune vs Endothelial* | *0.0011* | *2x2 fisher exact* |
| *Immune vs Fibroblast* | *0.1821* | *2x2 fisher exact* |
|  |  |  |
| *Epithelial vs Endothelial* | *0.8867* | *2x2 fisher exact* |
| *Epithelial vs Fibroblast* | *0.0008* | *2x2 fisher exact* |
| *Endothelial vs Fibroblast* | *0.0018* | *2x2 fisher exact* |

- p. 7, l. 40: Ablation study very interesting!

*We thank the reviewer for the comment.*

- p. 8, l. 22: Important to show, as the first thought that comes to mind is that these are cycling cells.

*It was also the first thing that came to our mind, and it surprised us when the signature did not associate with growth.*

- p. 8, l. 25: I would re-run these analyses without the cell-cycle related genes, especially as they are purported not to affect classification accuracy.

*We have re-run the SEEK co-expression search, GO analysis and the mSigDB enrichment analysis without the cell-cycle module. Both the GO, and the SEEK analysis did not return any significantly enriched terms.*

*The following figure shows the mSigDB enrichment results.*

*The enriched terms have not changed substantially, and still do not give an indication about a putative common biological function for the tumor gene signature.*

*To have consistency (we have used genes with the cell cycle module in all analysis in the paper), we have decided to include the enrichment results with the whole gene list, but have noted in the text changes resulting from the removal of the cell-cycle module from the enrichment analysis.*

- p. 8, l. 38: Why so low for colorectal cancer, which the gene set was derived from?

*We do not have a concrete explanation for why the tumor gene signature does not contain prognostic markers in the colorectal carcinoma.*

*We wondered whether colorectal cancer has a disproportionately low number of prognostic markers.*

*The following figure shows the number of prognostic genes per cancer type.*

*Colorectal cancer lies in the middle of distribution with marginally more genes related to the favorable prognosis, which means that the frequency of prognostic markers does not explain the discrepancy.*

*Our working hypothesis is that the CNVs do not contain strong prognostic information in colorectal cancers* [*(Chang, Miao, and Zhao 2019)*](https://paperpile.com/c/97637O/fQQH)*, which is undergoing investigation.*

- p. 10, l. 29: Missing space after colon.

*Corrected.*

- All subfigures have gigantic letters, they should be much smaller.

*We have now reduced the font on all subfigure letters..*

- Fig. 1: No statistics. This appears throughout the manuscript and in more places that I do not explicitly list.

*We have now included statistical tests for figures Fig.1D - F, and Fig. S1B. The results are reported in the figure legends.*

*We have, in addition created, a Supplementary Table 2 which contains a comprehensive list of statistical methods, types of tests, sample sizes, multiple test corrections, and summary statistics for each figure in the manuscript*

- Fig. 2: No statistics.

*We have now included statistical tests for figures Fig.2A, and B.*

- Fig. 2D: I see no Epithelial cells or Other cells (blue or purple), and the brown color is not in the legend

*We have corrected the figure legend.*

- Fig. 3A: No statistics.

*We have now included statistical tests for figures Fig.3A.*

- Missing S3

*Figure S3 was originally a table that was converted into Supplementary table - Copy of Effects of SAA1 and FGB. We have corrected the wrong designations in the text.*

*We have constructed a new Figure S3 from the previous Figure S2, while the new figure S2 contains perturbation experiments suggested by the reviewers.*

- Fig. S4: Too small, cannot read or interpret most of this figure.

*For subfigure A we have indicated that the comprehensive set of gene names can be found in the Supplementary Table 1 - Gene signatures, while we have indicated only the cell cycle genes in the figure.*

*For Subfigure 2 we have now increased the size of the text on the subfigure B, so that it is readable*

- I was unable to build Ikarus with the error "ModuleNotFoundError: No module named 'numpy'" from pyarrow, even though numpy is installed and is able to be loaded. To fix this, I must use "pip install -e . --no-use-pep517 --no-build-isolation", but this results in hanging with no progress on Python 3.9.6.

*We would like to thank the reviewer for noticing this error. Upon inspection we have determined that the error lies in an upstream dependency - the python package pySCENIC, which seems to be incompatible with python versions 3.9 or greater, due to conflicts in the numpy version requirements. We have notified the authors of the pySENIC package about the error.*

*We have now implemented a continuous integration using github workflows which tests the package build using multiple python versions. The package can also be installed directly through pip.*

The code itself had very few comments to help facilitate open-source collaboration.

*We have added extensive documentation to the code, have improved the verbosity of the notebook tutorial, and have added an example of how to run the package in R through reticulate.*

# References

[Abdelaal, Tamim, Lieke Michielsen, Davy Cats, Dylan Hoogduin, Hailiang Mei, Marcel J. T. Reinders, and Ahmed Mahfouz. 2019. “A Comparison of Automatic Cell Identification Methods for Single-Cell RNA Sequencing Data.” *Genome Biology* 20 (1): 194.](http://paperpile.com/b/97637O/J7iY)

[Chang, Zhiqiang, Xiuxiu Miao, and Wenyuan Zhao. 2019. “Identification of Prognostic Dosage-Sensitive Genes in Colorectal Cancer Based on Multi-Omics.” *Frontiers in Genetics* 10: 1310.](http://paperpile.com/b/97637O/fQQH)

[Hu, Jian, Xiangjie Li, Gang Hu, Yafei Lyu, Katalin Susztak, and Mingyao Li. 2020. “Iterative Transfer Learning with Neural Network for Clustering and Cell Type Classification in Single-Cell RNA-Seq Analysis.” *Nature Machine Intelligence* 2 (10): 607–18.](http://paperpile.com/b/97637O/WFDP)

[Jerby-Arnon, Livnat, Cyril Neftel, Marni E. Shore, Hannah R. Weisman, Nathan D. Mathewson, Matthew J. McBride, Brian Haas, et al. 2021. “Opposing Immune and Genetic Mechanisms Shape Oncogenic Programs in Synovial Sarcoma.” *Nature Medicine* 27 (2): 289–300.](http://paperpile.com/b/97637O/LJS3)

[Laughney, Ashley M., Jing Hu, Nathaniel R. Campbell, Samuel F. Bakhoum, Manu Setty, Vincent-Philippe Lavallée, Yubin Xie, et al. 2020. “Regenerative Lineages and Immune-Mediated Pruning in Lung Cancer Metastasis.” *Nature Medicine* 26 (2): 259–69.](http://paperpile.com/b/97637O/MJV7G)

[Lee, Hae-Ock, Yourae Hong, Hakki Emre Etlioglu, Yong Beom Cho, Valentina Pomella, Ben Van den Bosch, Jasper Vanhecke, et al. 2020. “Lineage-Dependent Gene Expression Programs Influence the Immune Landscape of Colorectal Cancer.” *Nature Genetics* 52 (6): 594–603.](http://paperpile.com/b/97637O/cTRfX)

[Li, Jia, Quanhu Sheng, Yu Shyr, and Qi Liu. 2022. “scMRMA: Single Cell Multiresolution Marker-Based Annotation.” *Nucleic Acids Research* 50 (2): e7.](http://paperpile.com/b/97637O/Z3FAl)

[Ma, Lichun, Limin Wang, Subreen A. Khatib, Ching-Wen Chang, Sophia Heinrich, Dana A. Dominguez, Marshonna Forgues, et al. 2021. “Single-Cell Atlas of Tumor Cell Evolution in Response to Therapy in Hepatocellular Carcinoma and Intrahepatic Cholangiocarcinoma.” *Journal of Hepatology* 75 (6): 1397–1408.](http://paperpile.com/b/97637O/oZoS)

[Michielsen, Lieke, Marcel J. T. Reinders, and Ahmed Mahfouz. 2021. “Hierarchical Progressive Learning of Cell Identities in Single-Cell Data.” *Nature Communications* 12 (1): 2799.](http://paperpile.com/b/97637O/DYn0)

[Puram, Sidharth V., Itay Tirosh, Anuraag S. Parikh, Anoop P. Patel, Keren Yizhak, Shawn Gillespie, Christopher Rodman, et al. 2017. “Single-Cell Transcriptomic Analysis of Primary and Metastatic Tumor Ecosystems in Head and Neck Cancer.” *Cell* 171 (7): 1611–24.e24.](http://paperpile.com/b/97637O/1Xj7)

[Wang, Lifei, Rui Nie, Zeyang Yu, Ruyue Xin, Caihong Zheng, Zhang Zhang, Jiang Zhang, and Jun Cai. 2020. “An Interpretable Deep-Learning Architecture of Capsule Networks for Identifying Cell-Type Gene Expression Programs from Single-Cell RNA-Sequencing Data.” *Nature Machine Intelligence* 2 (11): 693–703.](http://paperpile.com/b/97637O/sYRB)

[Zhang, Allen W., Ciara O’Flanagan, Elizabeth A. Chavez, Jamie L. P. Lim, Nicholas Ceglia, Andrew McPherson, Matt Wiens, et al. 2019. “Probabilistic Cell-Type Assignment of Single-Cell RNA-Seq for Tumor Microenvironment Profiling.” *Nature Methods* 16 (10): 1007–15.](http://paperpile.com/b/97637O/oNrQS)

**Second round of review**

**Reviewer 1**

1. The authors didn't compare with state-of-arts methods enough.
2. The title is too big as a scholar paper. I cannot see the novelty from the title.

**Reviewer 2**

The authors have done a fantastic job addressing my comments, and I recommend this manuscript for publication.
